# Effect of Steelmaking Slag Additives on Mullitization and Phase Composition of Chamotte Refractories

**DOI:** 10.3390/ma19122438

**Published:** 2026-06-07

**Authors:** Saniya Arinova, Svetlana Kvon, Vitaliy Kulikov, Assem Altynova, Nurdaulet Zharylkassin

**Affiliations:** Department of Metallurgy and New Materials, Abylkas Saginov Karaganda Technical University, Nazarbayev Avenue, No. 56, Karaganda 100027, Kazakhstan; s.arinova@ktu.edu.kz (S.A.); v.kulikov@ktu.edu.kz (V.K.); aasemaa@mail.ru (A.A.); nurdaulet21042003@gmail.com (N.Z.)

**Keywords:** chamotte refractories, steelmaking slag, mullitization, clinoferrisilite, optical dilatometry, thermal stability, utilization of technogenic waste

## Abstract

Steelmaking produces large volumes of slag, a by-product with environmental risks due to accumulation and possible contamination. This study explores its use as a mineralizing agent in chamotte refractories. Slag rich in clinoferrisilite was added up to 5 wt.% to partially replace fine chamotte. Samples were shaped by semi-dry pressing and fired at 1350 °C. Chemical and phase composition, thermal behavior, microstructure, and physico-mechanical properties were analyzed. Results showed slag addition increased mullite content to 68 wt.% and promoted secondary magnesium–aluminosilicate phases (indialite, cordierite), indicating activation of reactions in the MgO-Al_2_O_3_-SiO_2_ system. DSC and TGA revealed thermal effects between 1298 and 1325 °C, confirming slag’s fluxing role and lowering the liquid-phase sintering temperature. Optimal properties were achieved with 5% slag and 10% clay, yielding compressive strength of 24 MPa and apparent density of 2.30 g/cm^3^, meeting GOST 390-96 requirements for grade SHA. However, excess liquid-phase components reduce thermal stability. Thus, steelmaking slag is an effective secondary raw material, enhancing mullitization and refractory performance when used within controlled limits.

## 1. Introduction

The rapid industrial development is accompanied by the accumulation of substantial volumes of industrial waste, resulting in significant environmental and socio-economic repercussions. The disposal of solid industrial waste represents one of the central challenges for sustainable development, particularly for countries with emerging economies. A considerable proportion of such waste is composed of by-products from metallurgical processes, including steelmaking slags and fine particulate matter from gas cleaning systems.

Approximately 32 billion tons of waste have been accumulated in the Republic of Kazakhstan, with over 16 billion tons being of technogenic origin. Each year, around 700 million tons of industrial waste are generated [1]. A significant portion of this waste is associated with the ferrous metallurgy industry. The disposal of metallurgical slags in open landfills leads to the migration of harmful substances into the soil and groundwater. This creates a potential environmental threat and poses risks to public health.

The reuse of metallurgical waste as secondary raw materials for production of construction and refractory materials is an effective approach to reducing environmental impact and conserving resources. However, the degree of utilization of steelmaking slag remains low [2,3]. This is due to the complex phase composition and heterogeneity of the slag. Nevertheless, slags possess significant potential as a source of silicon, aluminum, magnesium, and iron oxides. These oxides can participate in the formation of heat-resistant phases [4].

Chamotte refractories are aluminosilicate ceramic materials. The dominant phases in these materials are mullite (3Al_2_O_3_·2SiO_2_) and silica modifications, such as cristobalite and quartz. They are widely used in the metallurgical, glass, and cement industries due to their high thermal stability, chemical resistance, and cost-effectiveness [4,5]. The key factor determining the operational characteristics of these materials is their phase composition and microstructure. These are formed during high-temperature firing.

Chamotte compositions are traditionally produced from aluminosilicate components, such as chamotte scrap, kaolin, and quartz. This is done through shaping and high-temperature firing, typically in the range of 1300–1550 °C. The goal is to form a mullite crystalline matrix and to distribute silica phases within the structure [6,7]. The formation of mullite is a key process. It begins at temperatures around 1000 °C and is accompanied by sequential phase transitions of the clay components. An increase in mullite content generally leads to improved strength, thermal stability, and creep resistance. However, silica phases can have both positive and negative effects on the material properties. This is due to the specific characteristics of their phase transitions and distribution within the structure [4,5].

The phase composition of chamotte materials directly determines their physicomechanical properties. The proportion of mullite in fired products correlates with mechanical strength and thermal cycling resistance. Higher mullite content is generally associated with improved strength, better resistance to thermal stresses, and reduced susceptibility to creep at high temperatures [8,9,10]. At the same time, silica phases can have a dual effect. Residual quartz can decrease resistance to thermal stresses due to low-temperature phase transitions. In contrast, cristobalite, which forms during firing above approximately 1250 °C, can enhance refractoriness. However, it also increases sensitivity to thermal cycling due to the α-β phase transition of SiO_2_ [6].

A longstanding issue in the global scientific literature is the study of phase transformations and their influence on microstructure and performance properties. This is often explored within Al_2_O_3_-SiO_2_ systems. However, the incorporation of technogenic components, such as steelmaking slags, remains underexplored. Metallurgical slags contain significant amounts of silicon, magnesium, and iron oxides. These oxides can participate in the formation of high-temperature phases. Despite this, their use in chamotte compositions is rarely discussed in the literature. Comprehensive reviews on this topic are almost nonexistent [11,12]. Some studies on the incorporation of secondary materials into refractories suggest that such additives can promote the formation of additional phases, such as magnesium–aluminosilicates or ferrosilicates. These additives can also influence the microstructure. However, there is a lack of consistent quantitative assessments regarding the effect of their content and nature on the final properties of the materials [13].

The study in related fields confirms that controlling phase composition and microstructure is critical for the functional properties of refractories. For instance, studies showed that increasing the mullite content in mullite-zircon-based refractories positively impacted their mechanical properties and corrosion resistance. This effect is associated with the formation of a denser silica-alumina matrix [14]. Similarly, a study on the composition modification of refractory castings emphasizes the role of the phase evolution pathway. This factor influences the thickness and distribution of phases, which is crucial for predicting material behavior during heating [15].

Moreover, studies on the modification of refractories with magnesium-containing phases show that the inclusion of MgO and similar components in the system can promote the formation of secondary heat-resistant phases. These phases enhance thermal shock resistance and increase mechanical strength [16]. This conclusion aligns with empirical findings from the use of metallurgical slags enriched with Mg and Fe oxide components. Such components can play a structure-modifying role in refractories [13].

Despite the significant amount of the study on aluminosilicate refractories, the influence of technogenic additives, particularly steelmaking slags, on phase formation processes and microstructure development has not been fully studied. Steelmaking slags contain considerable amounts of iron, silicon, and magnesium oxides. These oxides can act as mineralizers and influence both solid-phase and liquid-phase sintering mechanisms. However, the mechanisms by which they affect mullitization, pore structure formation, and the behavior of refractories at high temperatures remain insufficiently understood.

Most existing studies focus either on the analysis of phase composition or on the evaluation of mechanical properties. Comprehensive examinations of the relationship between “thermal behavior, phase formation, microstructure, and properties” are limited. In particular, the role of ferrosilicate phases in the formation of the liquid phase requires further study. Additionally, their impact on the operational characteristics of refractories remains insufficiently explored.

The aim of this study is to investigate the effect of steelmaking slag on phase formation and microstructure in chamotte refractories. Additionally, the impact on the physicomechanical properties is examined, with particular focus on liquid-phase sintering processes.

## 2. Materials and Methods

Three main components were used to produce the chamotte refractory products: chamotte scrap, kaolin clay, and steelmaking slag. The slag was provided by the Karaganda Machine-Building Plant named after Pakhomenko (Karaganda) and was obtained during steel production. The choice of these components was based on their aluminosilicate nature. Additionally, their ability to contribute to the formation of high-temperature mullite phases during the firing process was considered.

The chemical composition of the starting components was determined by X-ray fluorescence spectrometry. A multi-channel analyzer, ARL QUANT’X, was used for the analysis. This method enabled the determination of the content of the major oxides (Table 1).

**Figure 1:**
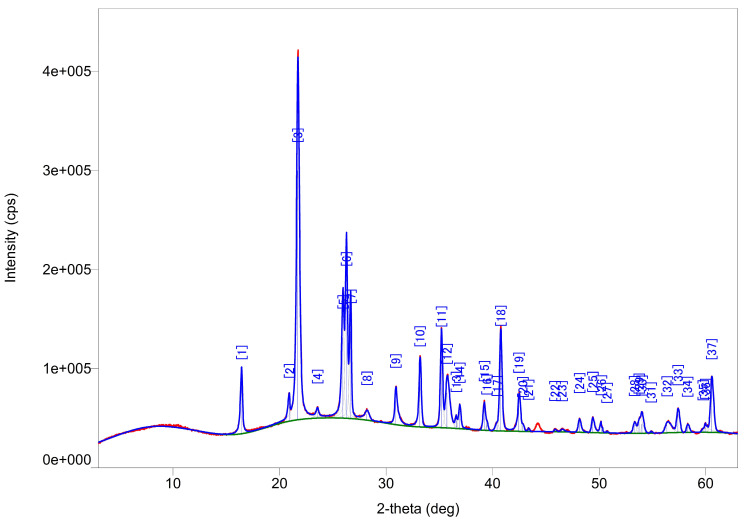
XRD pattern of chamotte scrap; numbers [1]–[37] correspond to the serial numbers of the fitted diffraction peaks arranged in order of increasing 2θ.

X-ray phase analysis (XPA) of the raw samples was performed to identify the main crystalline phases in chamotte and clay (Figure 1, Table 2). Silicate compounds in the slag, acting as mineralizers, were also identified.

Chamotte serves as the main filler. It provides high thermal resistance due to its high mullite content. Quartz and cristobalite contribute to the formation of the silicate matrix.

**Figure 2:**
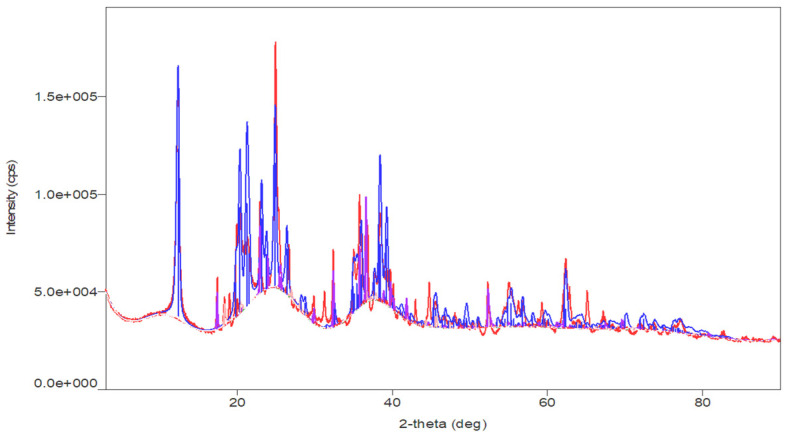
Diffractogram of clay.

The raw material used was clay from the Krasnooktyabrskoye deposit (Kostanay region, Kazakhstan) (Figure 2, Table 3). This clay served as the binding component and a source of aluminosilicates.

As shown in the data, kaolinite constitutes the major part of the mineral composition. The presence of kaolinite is a characteristic feature of refractory clays used in the production of chamotte materials.

It is well known that kaolinite, when heated above 500 °C, transforms into metakaolinite. This phase then reacts with silica to form a crystalline mullite matrix at temperatures between 1300 and 1350 °C [11].

A significant portion of the phase composition is made up of forsterite, which constitutes approximately 23%. This magnesium-containing silicate is characteristic of magnesium-enriched aluminosilicate systems. Its presence may indicate the inclusion of magnesium-containing minerals in the raw material. Alternatively, it could reflect a natural characteristic of the deposit. Forsterite is known for its high refractoriness and thermal stability. Therefore, its presence may positively impact the thermal resistance of the material.

It is also important to note that the presence of magnesia and high-alumina minerals in the clay creates favorable conditions for the formation of a mullite-cordierite structure. However, the high concentration of active impurities in the clay necessitates careful dosing of flux-containing additives. This is essential to preserve the required refractoriness of the material.

Slag is used as a secondary component and a mineralizer that accelerates crystallization. The main phases include clinoferrisilite (dominant), cristobalite, and quartz. The slag facilitates the formation of magnesium–aluminosilicate phases, improving both thermal resistance and mechanical strength of the chamotte bricks [17,18].

A comprehensive phase analysis of the raw components confirmed their functional role in the production of the refractory material. Chamotte scrap acts as the main structural framework, ensuring the presence of primary mullite, which constitutes 59.8%. The binder is kaolin clay, containing 70% kaolinite. Quartz (23%) and magnesium microimpurities (chlorites) in the clay create conditions for the formation of secondary mullite and magnesium–aluminosilicate phases during firing.

**Figure 3:**
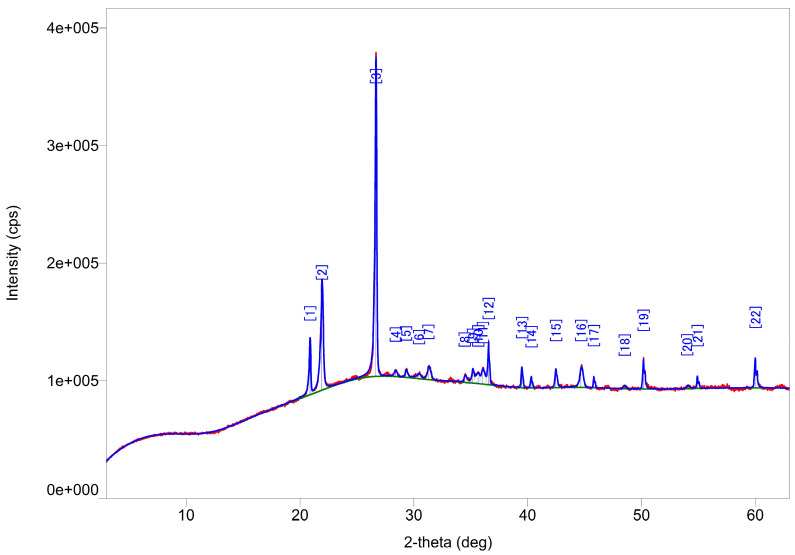
XRD pattern of slag; numbers [1]–[22] correspond to the serial numbers of the fitted diffraction peaks arranged in order of increasing 2θ.

The introduction of steelmaking slag, predominantly composed of clinoferrisilite (FeSiO_3_—81%), facilitates intense mineralization of the system (Figure 3, Table 4). The presence of this multi-component ferrosilicate system results in high reactivity and a tendency for liquid-phase sintering. Furthermore, the inclusion of calcium- and magnesium-silicates promotes structural modification. This may potentially enhance both the density and thermal stability of the materials being formed.

Five formulations (Nos. 1–5) were developed during the experiment. The clay content varied from 10% to 15%, while the slag content ranged from 0% to 5%. The proportion of the structural framework remained unchanged. This approach enabled the assessment of the impact of mineralizing additives on the structural integrity of the refractory material.

### 2.1. Preparation of the Charge

**Figure 4:**
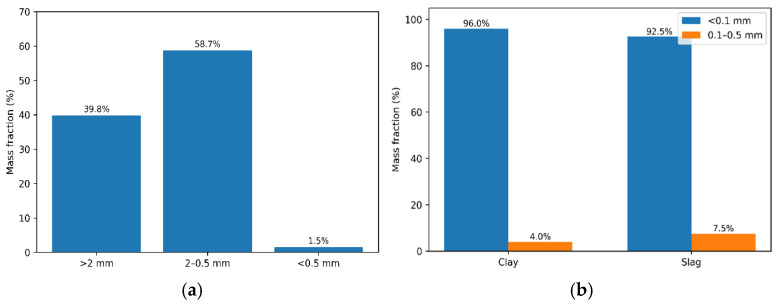
Particle size distribution of raw materials: (**a**) chamotte; (**b**) clay and slag.

The filler preparation included crushing and sieving. Sieving was performed using sieve analysis according to GOST 3584.1 [18]. The particle size distribution was as follows: >2 mm—39.8%, 2–0.5 mm—58.7%, and <0.5 mm—1.5% (Figure 4). This distribution was selected based on literature data [19,20,21]. It was shown that large particles (>2 mm) form a strong framework and reduce shrinkage during firing. Medium particles (2–0.5 mm) fill the intergranular spaces, increasing density. Fine particles (<0.5 mm) promote sintering and facilitate bonding between grains.

The fractional composition of the chamotte mixture ensures optimal strength and thermal resistance of the refractories. Large chamotte particles form the framework. Smaller particles fill the intergranular spaces. Clay particles (<0.1 mm) actively participate in mullite formation. Fine slag mineralizes the mixture, enhancing mechanical stability. This distribution reduces porosity and prevents crack formation. It also ensures uniform stress redistribution during heating. The optimal ratio of fractions improves both mechanical properties and thermal resistance [9].

The compositions of the charge for samples Nos. 1–5 are presented in Table 5.

### 2.2. Manufacturing Technology

The samples were formed using the semi-dry pressing method. Water addition ranged from 5.5% to 6.5% by weight. This ensured the necessary plasticity of the mixture and facilitated uniform pressing. Semi-dry pressing was selected as it minimizes shrinkage and the risk of cracking. Additionally, it ensured a homogeneous pore structure, which was crucial for analyzing the effect of phase composition on strength [22].

**Figure 5:**
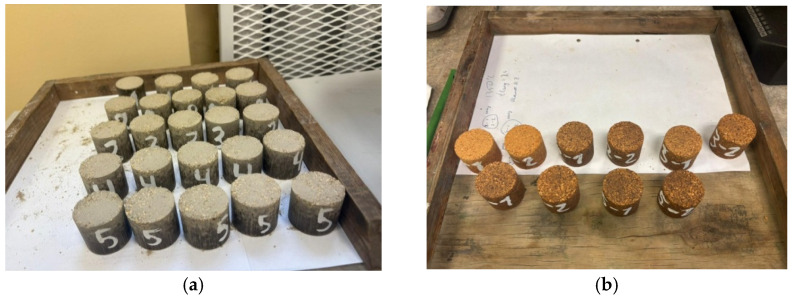
General view of the samples. (**a**) Pressed samples; (**b**) Samples after firing at 1350 °C.

For physicomechanical experiment, cylindrical samples with a diameter of 30 mm and a height of 50 mm were produced. A manual press (RP-50) was used, applying a uniaxial load of 50 kN (Figure 5a).

The samples were dried at 105 ± 5 °C until a constant weight was achieved. Following this, they were fired in a laboratory electric furnace using a programmed heating schedule. The temperature was raised at 5 °C/min to 600 °C, and then at 3 °C/min to 1350 °C, with a holding time of 2 h (Figure 4b). A temperature of 1350 °C ensured the completion of clay dehydroxylation. It also promoted the formation of a stable mullite structure without any melting [11,17,18].

### 2.3. Study of Sample Properties

Open porosity, apparent density, and water absorption were determined by the water saturation method in accordance with GOST 2409-2014 [23]. The parameters were calculated using standard relationships, taking into account the mass of the dry, water-saturated, and liquid-weighed samples.

For distilled water, the liquid density was ρ*l* = 1 g/cm^3^. In the case of using kerosene (if the experimental material reacts with water), the liquid density was ρ*l* = 0.789 g/cm^3^.

Compressive strength tests were performed using a laboratory testing press (IP-50). The load was applied in a direction perpendicular to the working surface of the sample. The load was gradually increased until signs of failure appeared. The load range was from 1 to 50 tons (tc), with a measurement error not exceeding 1%. Several samples of each composition were tested. The average value of the obtained results was taken as the final outcome.

The compressive strength limit of refractory materials was determined in accordance with the requirements of GOST 4071.1–2021 “Refractory products with total porosity less than 45%. Method of determining the compressive strength limit at room temperature”. The test involved recording the maximum uniaxial compressive load applied to the sample until failure occurred. The measured force was then recalculated based on the cross-sectional area of the sample.

The softening temperature of the experimental materials was determined using a visual sintering temperature analyzer, SJY-II-17 (Hunan Zhenhua Analysis Instrument Co., Ltd., Xiangtan, China) (Imaging Sintering Point Tester). This device is a high-temperature optical dilatometer. It allows for the recording of shape changes in the sample during heating. Additionally, it enables the identification of temperatures corresponding to key stages of the material’s thermal behavior.

The study of thermal processes in the experimental compositions was conducted using differential thermal analysis (DTA) with the Thermoscan-2 setup. This instrument was employed to determine temperature intervals and to quantitatively assess thermal effects associated with endothermic and exothermic reactions in the material. Measurements were performed in the temperature range from 25 °C (room temperature) to 1000 °C. The setup allowed for variation in the heating rate from 0.5 to 20 °C/min. This enabled flexible adjustment and accurate recording of the dehydration stages, as well as the initial mineralization processes that form the structure of the refractory material.

Additionally, to assess the thermal behavior of the original steelmaking slag, DSC-TGA analysis was conducted under similar conditions. This method enabled the determination of temperature intervals for phase transitions. It also provided insights into the potential formation of a liquid phase, which influences the sintering processes.

The phase composition of the raw material and fired samples was determined using X-ray diffraction (XRD) analysis on a Rigaku Miniflex 600 diffractometer (Rigaku, Tokyo, Japan). The analysis was conducted with CuKα radiation, using a scanning step of 0.02°. Instrument control and initial data processing were performed with the MiniFlex Guidance software. The subsequent analysis was carried out using the PDXL Basic package. Phase identification was achieved by comparing the experimental diffraction peaks with data from the JCPDS database.

The surface morphology and sintering efficiency were studied using a JEOL JSM-7001F scanning electron microscope (JEOL Ltd., Tokyo, Japan). To enhance surface conductivity, the samples were coated with a thin layer of carbon. This coating was applied using a Q150T ES Plus sputter coater (Quorum Technologies Ltd., Lewes, UK).

Thixomet PRO software version 6.4 was used for microstructural analysis, enabling semi-automatic segmentation of SEM images. Using this approach, pores and phase regions were identified, followed by the calculation of quantitative parameters, including pore phase fraction, total pore area, average equivalent pore size, and phase area fraction.

## 3. Results

All chamotte samples comply with the requirements of grade SHA according to GOST 390-96. The open porosity ranges from 21% to 24%. Water absorption is between 2.0% and 2.7%. The compressive strength varies from 19 to 24 MPa (Table 6). Sample No. 2 is the most optimal based on the combination of properties. It demonstrates high porosity, density, water absorption, and compressive strength. The other samples also meet the regulatory requirements. However, their compressive strength and water absorption are slightly lower. In general, all samples are suitable for use in SHA-grade refractory constructions [8].

The highest compressive strength of 24 MPa was observed in sample No. 2, despite its relatively high porosity.

This fact indicates that the mechanical strength of chamotte refractories is determined not only by the total porosity but also by its spatial organization.

For further studies, sample No. 2 was selected. It demonstrated the most optimal physicomechanical properties among all the experimental samples. The sample had the highest porosity of 24.2%. This provided good thermal insulation and structural stability at high temperatures. Its density of 2.30 g/cm^3^ and compressive strength of 24 MPa were the highest among all the samples. This indicated high mechanical stability and reliability of the material. The water absorption of 2.7%, although slightly elevated, remained within the permissible limits and corresponded to the high porosity.

The operational reliability of the developed materials was additionally confirmed by slag resistance tests. These experiments made it possible to evaluate the resistance of the refractories to chemical interaction with metallurgical slag. The results showed that the area of slag–refractory interaction varied within 127.24–141.97 mm^2^ (Table 7), while the best performance was demonstrated by sample No. 2 containing 5 wt.% slag, whose corrosion resistance was nearly identical to that of the reference chamotte composition. The average slag penetration depth was only 0.1–0.4 mm, indicating the formation of a dense protective barrier during high-temperature firing.

**Figure 6:**
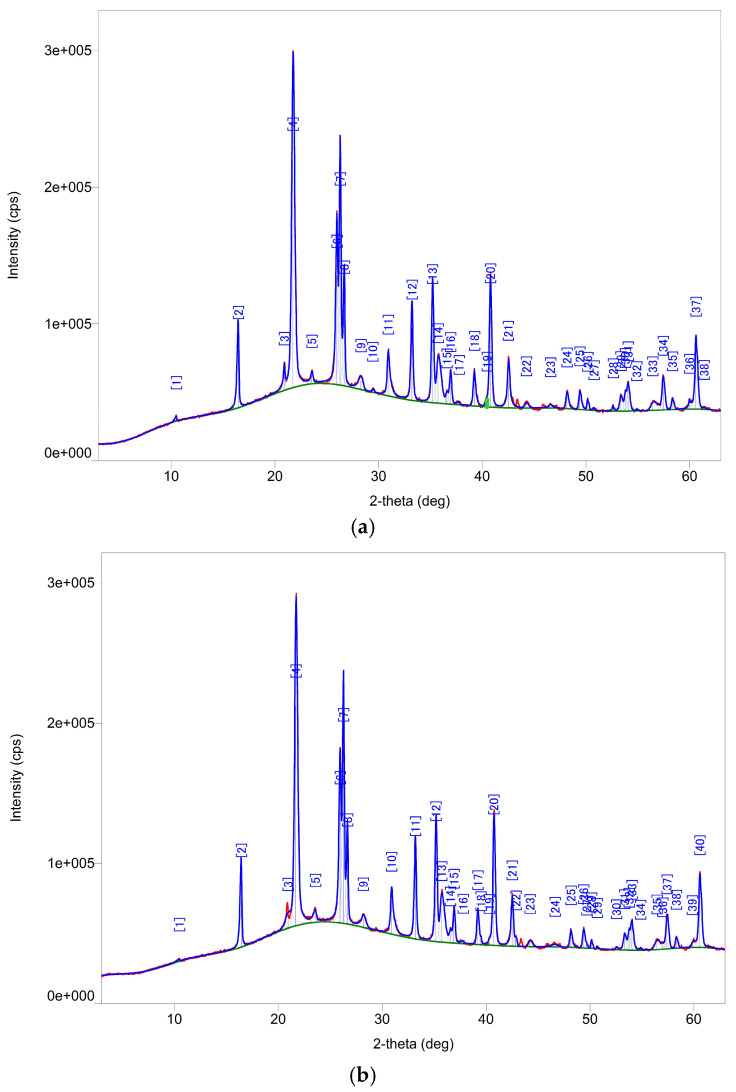

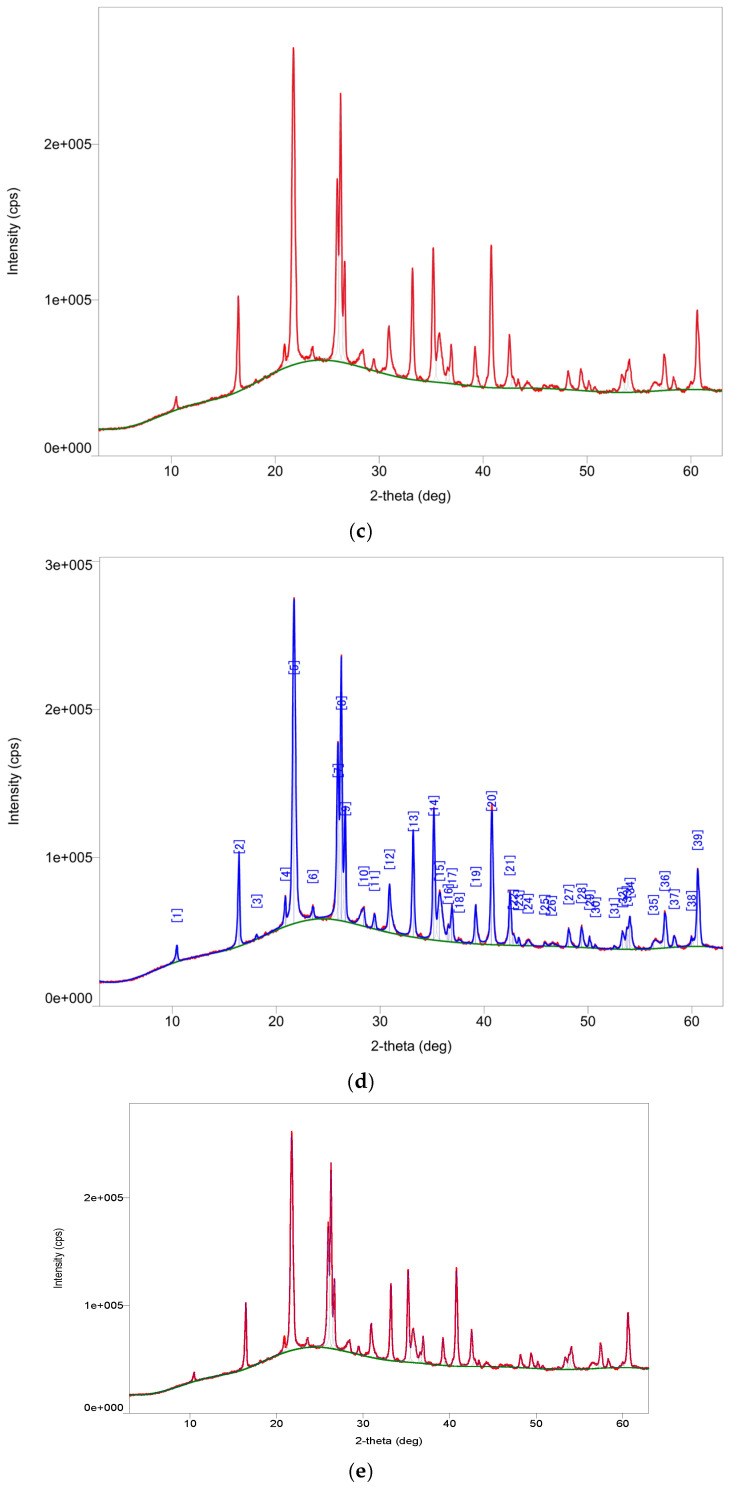
XRD pattern of xperimental samples after firing: (**a**)—No. 1; (**b**)—No. 2; (**c**)—No. 3; (**d**)—No. 4; (**e**)—No. 5. Peak numbers correspond to the serial numbers of the fitted diffraction peaks arranged in order of increasing 2θ.

To determine the phase composition of the experimental samples after firing, X-ray diffraction (XRD) analysis was conducted. The results of the XRD analysis are presented in Figure 6 and Table 8.

The study of the phase composition of the obtained refractories after firing at 1350 °C confirmed the formation of a developed crystalline aluminosilicate structure. X-ray diffraction (XRD) analysis revealed that mullite is the dominant phase in all the experimental samples. The content of mullite ranges from 62% to 68%. This high degree of mullitization confirms the correctness of the selected temperature-time firing regime. It ensures the completeness of solid-state reactions in the Al_2_O_3_–SiO_2_ system. The formed mullite framework is the primary factor determining refractoriness, mechanical strength, and dimensional stability at high temperatures.

The silica component of the materials is primarily represented by cristobalite, which constitutes 23–26%, and residual quartz, which constitutes 6–8%. The presence of a significant amount of cristobalite indicates the completion of high-temperature recrystallization processes of silicon dioxide. Meanwhile, the retention of a small amount of quartz is typical for chamotte systems. This suggests the heterogeneity of the initial raw material, where some of the filler grains serve as an inert framework.

Particular interest lies in the identification of secondary magnesium–aluminosilicate phases, such as indialite, cordierite-like compounds, and aluminous enstatite. Their formation in the experimental compositions No. 2–No. 5 is directly linked to the introduction of steelmaking slag. This slag is rich in clinoferrisilite (92%) and contains magnesium-bearing components. Additionally, the presence of magnesium in the kaolin clay contributes to the formation of these phases.

Even at relatively low contents of 0.6–8%, cordierite and indialite phases perform a structural-modifying function. They contribute to the densification of the ceramic matrix by intensifying liquid-phase sintering. This process involves the participation of the iron silicate phase identified in the slag composition and the reaction phase formation in the MgO-Al_2_O_3_-SiO_2_ system. The formation of a limited amount of liquid phase accelerates diffusion processes. This results in a denser packing of grains. These combined effects may reduce the thermal expansion coefficient and enhance the thermal stability of the refractory material.

Analysis of the diffractograms showed the absence of distinct peaks from low-temperature clay minerals. This confirmed the completeness of the thermal destruction of the initial raw material. The minimal content of the glassy amorphous phase suggested a predominantly crystalline nature of the formed structure. This is indicative of high-quality sintering. Thus, steelmaking slag functions not only as filler but also as an active mineralizer. It intensifies the growth of mullite crystals and aids in the formation of a dense, thermally stable microstructure.

**Figure 7:**
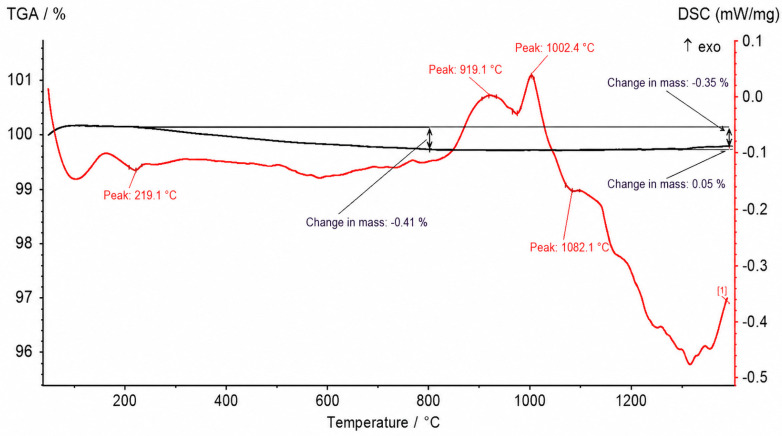
DSC-TGA curves of the steelmaking slag.

The DSC-TGA analysis of the steelmaking slag (Figure 7) reveals several thermal effects. One of these effects occurs in the high-temperature interval of 1298–1325 °C. This is likely associated with the formation of a liquid phase. The results confirm the fluxing action of the slag. They also demonstrate the slag’s ability to intensify sintering processes in the chamotte system.

The X-ray diffraction analysis of the fired samples confirmed the intensification of phase formation processes upon the introduction of steelmaking slag. It was established that the content of the main strengthening phase, mullite, increased to 68.0%. This value exceeded that of the reference sample (65.9%).

A distinctive feature of the modified compositions was the appearance of secondary magnesium-containing phases. These phases included indialite and high-temperature cordierite. The formation of these minerals was caused by the interaction of slag components with the products of thermal decomposition of the clay binder (metakaolin) at temperatures above 1100 °C. The reduction in the content of residual quartz in the experimental samples indicates the completeness of the sintering reactions. This suggests the formation of a more uniform and well-developed microstructure, which is crucial for improving mechanical strength and operational reliability. Primarily, the low thermal expansion of cordierite minerals contributes to the material’s high thermal stability. It effectively resists sharp temperature fluctuations. Furthermore, the substitution of residual quartz with cordierite increases the structural stability of the product. This substitution eliminates the risk of microcracking, which typically occurs due to volume changes in silicon dioxide.

**Figure 8:**
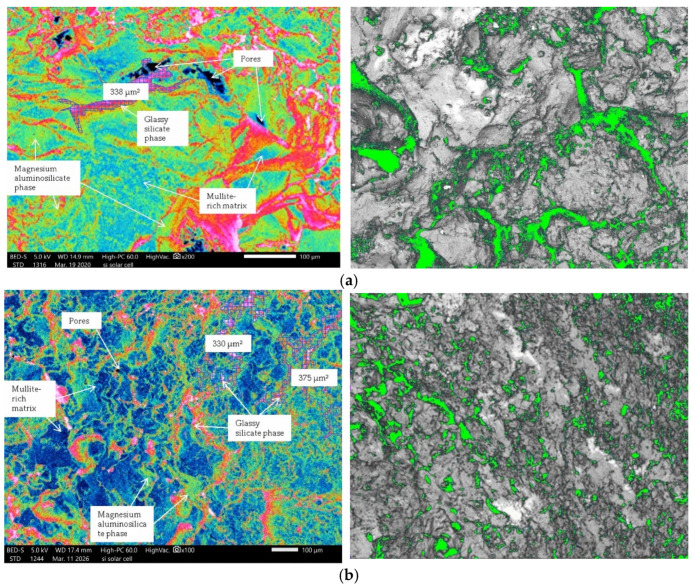

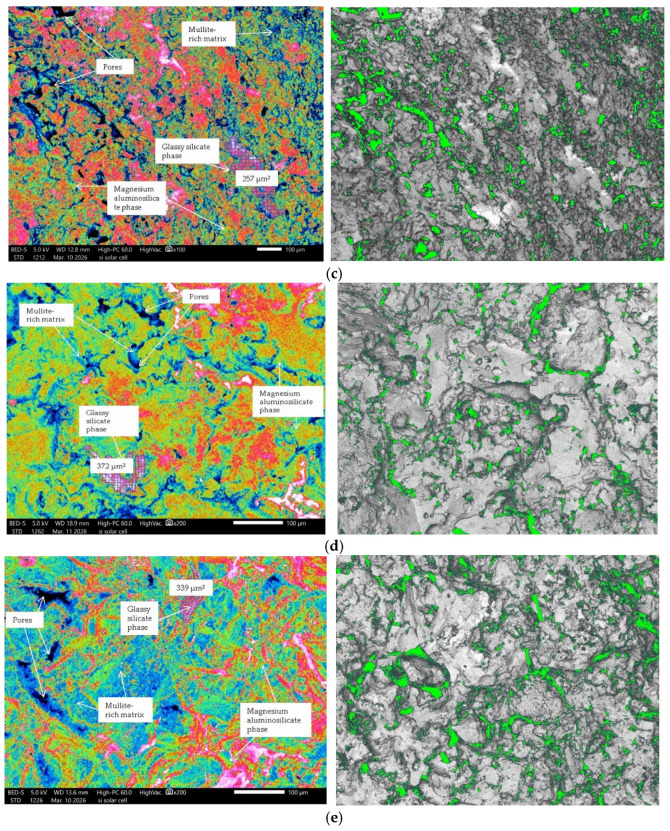
Microstructure of the fired samples: (**a**)—sample No. 1; (**b**)—sample No. 2; (**c**)—sample No. 3; (**d**)—sample No. 4; (**e**)—sample No. 5.

The microstructural investigation was supplemented by semi-automatic segmentation of SEM images using Thixomet PRO software version 6.4 (Figure 8, Table 9). This approach made it possible to determine quantitative parameters of the pore structure, including the pore phase fraction (%), total pore area (μm^2^), and average equivalent pore size. It should be emphasized that the obtained data characterize the two-dimensional pore area fraction on the polished section surface rather than the absolute volumetric porosity of the material.

The analysis results revealed significant differences between the investigated samples. Sample No. 2 demonstrated the most uniform distribution of fine pores, with an average pore size of 5.7 μm and the minimum pore phase fraction of 13.4%, indicating structural densification while preserving intergranular channels. In contrast, samples with a higher pore fraction (up to 34%) were characterized by the presence of extended intergranular pore channels and a less compact structure.

Comparison of the quantitative microstructural data with the physicomechanical characteristics confirmed a direct relationship between pore morphology and material properties. Samples with a uniform distribution of fine pores exhibited higher apparent density and compressive strength. In particular, sample No. 2, possessing the most homogeneous microstructure, demonstrated the maximum density (2.30 g/cm^3^) and compressive strength (24 MPa). An increase in pore area and pore-channel continuity was accompanied by a decrease in density and deterioration of mechanical properties.

Sample No. 1 exhibited the highest pore phase fraction (34%) and large pores with an average size of 14.6 μm, reflecting a loose structure and explaining its comparatively lower strength characteristics. In contrast, sample No. 2 was characterized by minimal porosity (13.4%) and a uniform distribution of fine pores (~5.7 μm). Such a compact structure is directly associated with its maximum density (2.30 g/cm^3^) and compressive strength (24 MPa).

In samples No. 3–5, the pore fraction remained at the level of 20–21%; however, the formation of extended intergranular pore channels was observed. This resulted in a less densified structure and reduced mechanical stability.

**Figure 9:**
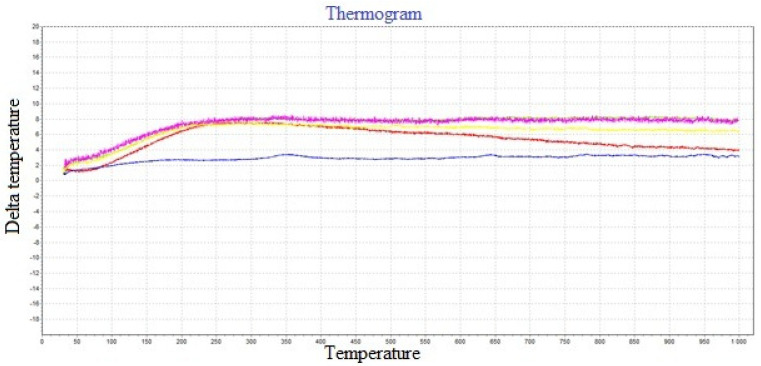
Thermogram: No. 1—red, No. 2—blue, No. 3—green, No. 4—pink, No. 5—yellow.

Based on the data on the mineral composition and microstructure of the samples, their thermal stability upon repeated heating was assessed. All compositions were examined using high-temperature differential thermography up to 1000 °C (Figure 9). This method confirmed the absence of hidden phase transitions or changes in the formed structure during operation.

The results of the thermal analysis, conducted within the temperature range up to 1000 °C, complement the X-ray diffraction data. These results provide information on the phase stability of the obtained materials. The thermograms for all compositions show no distinct endothermic or exothermic effects in the high-temperature region. This confirms the completion of the main processes, including dehydroxylation of clay minerals and the formation of the mullite structure during the preliminary firing at 1350 °C.

It should be noted that, unlike the other samples, sample No. 2 (blue line) shows a reduction in the thermal effect. This may be due to a lower degree of liquid phase formation or more complete sintering processes in this sample.

Thus, the thermoscanning data confirm the high operational reliability of the developed refractories. They also confirm the stability of their phase composition under conditions of intense heating. The obtained results correlate with the X-ray diffraction data. They suggest that the selected thermal treatment regime and the introduction of an active iron-bearing mineralizer contribute to the formation of a thermally inert and mechanically strong material structure.

The mullite content in sample No. 2 (67 wt.% at 1350 °C) is located at the upper limit of the typical range for SHA-grade chamotte refractories (45–70 wt.% [24]) and is comparable with the results reported for refractories produced using secondary raw materials. For example, the addition of 5 wt.% spent magnesia–carbon refractories was reported to yield approximately 69.9% mullite at 1300 °C, followed by a decrease to 65.9% at 1400 °C due to cordierite formation [25]. The present results confirm a common phase-formation mechanism in MgO-containing systems.

The obtained materials also demonstrate competitive mechanical properties: the compressive strength reached 24 MPa at a porosity of 25.2%. According to the literature, the addition of alumina-containing slags up to 5 wt.% improves strength due to their mineralizing effect [26]. In the present study, high strength was achieved without reducing porosity, which represents a technological advantage.

The content of secondary phases, such as indialite (~0.6%), remained minimal compared with previously reported systems (3–5%), allowing the dominant role of mullite to be preserved and preventing weakening of the ceramic matrix. Thus, composition No. 2 demonstrates an optimal combination of high strength, porosity, and slag resistance, exceeding the performance of many previously reported modified chamotte refractories.

The confirmed phase stability and the absence of hidden transformations upon heating up to 1000 °C enable the evaluation of the ultimate operational capabilities of the developed refractories. A critical factor for materials of this type is their behavior in the range of critical temperatures. This is the temperature range where softening and deformation begin due to the material’s own weight.

**Figure 10:**
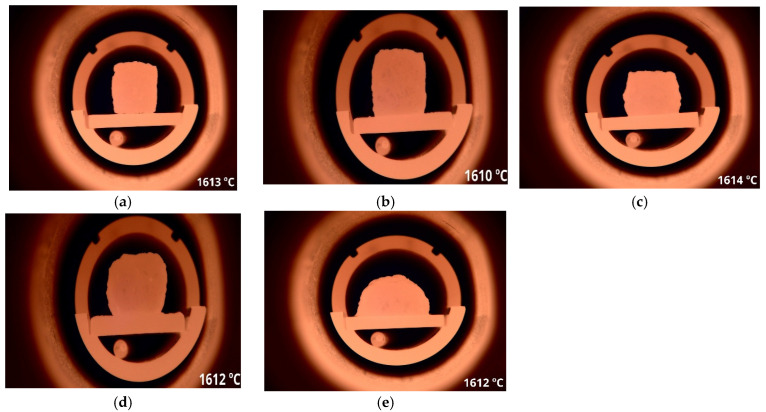
Shape changes in the studied samples during heating in the sintering point analyzer: (**a**)—sample No. 1; (**b**)—sample No. 2; (**c**)—sample No. 3; (**d**)—sample No. 4; (**e**)—sample No. 5.

The deformation onset temperature was determined using optical analysis with the SJY-II-17 instrument. Continuous image registration during programmable heating allowed for the identification of the characteristic deformation stages of the samples (Figure 10). It was observed that the changes in their geometric parameters at temperatures ranging from 1610 °C to 1614 °C correspond to the high thermal stability of the selected compositions.

Samples No. 1 and No. 2 retained almost unchanged geometric shapes when heated up to 1613 °C. Only slight contour refinement was observed. This was caused by thermal expansion and the initial stage of particle sintering. No signs of intense softening or transition to a viscoplastic state were detected within this temperature range.

For samples No. 3, No. 4, and No. 5, intense deformation was observed to begin at temperatures around 1612–1613 °C. At this stage, noticeable changes in geometry were observed. These changes included rounding of the upper part, a decrease in height, and an increase in the base area. Such behavior indicates the transition of the material into a viscoplastic state. This marks the beginning of structural softening, which is accompanied by a natural decrease in mechanical stability at high temperatures.

Sample No. 5, despite exhibiting the highest degree of mullitization (68%) and the formation of the cordierite phase, which typically enhances strength, showed a lower deformation onset temperature during the experiment. This phenomenon can be attributed to the specific chemical composition of the slag used. According to classical sintering theory, an increased content of iron-bearing components above 1500 °C promotes the formation of an excessive amount of low-viscosity liquid phase. This melt acts as a “lubricant” between mullite crystals. As a result, the material transitions prematurely into a viscoplastic state.

Based on a comprehensive comparison of strength characteristics and thermal stability, it can be concluded that composition No. 2 is the most balanced. The 5% slag addition ensures a high mechanical strength of 24 MPa, due to active mullitization (67%). At the same time, the composition retains the necessary thermal shape stability. It prevents excessive glass formation under extreme loading conditions.

## 4. Conclusions

The conducted study confirmed the effectiveness of using up to 5 wt.% steelmaking slag in chamotte refractories. It also demonstrated its significant impact on phase and structural formation processes. It was found that the dominant phase in all compositions is mullite (62–68 wt.%), which forms the supporting framework of the material. The introduction of slag has a fluxing effect. It intensifies mullitization up to 68 wt.% and promotes the formation of secondary magnesium–aluminosilicate phases, such as indialite and cordierite.

DSC-TGA analysis confirmed a reduction in the liquid-phase sintering temperature to 1298–1325 °C. The thermal stability of the fired samples was maintained up to 1000 °C. Microstructural analysis revealed the formation of an intergranular glass phase. It also showed densification of the ceramic matrix.

Optimal properties were observed for composition No. 2 (5% slag, 10% clay). This composition exhibited a compressive strength of 24 MPa and a density of 2.30 g/cm^3^. Deviations from the optimal formulation resulted in either structural heterogeneity (sample No. 3) or the formation of an excessive liquid phase (sample No. 5). These effects led to a reduction in thermal resistance at temperatures above 1613 °C.

Thus, the influence of steelmaking slag is dual. On one hand, it promotes the intensification of mullitization and densification of the structure. On the other hand, when the slag content exceeds the optimal level, it leads to a reduction in high-temperature stability. The optimal slag content is approximately 5 wt.%.

The developed approach improves the quality of chamotte refractories. It also facilitates the efficient utilization of metallurgical waste. This method allows for the partial replacement of natural raw materials with technogenic ones. Importantly, this substitution does not compromise the material’s operational properties. As a result, it contributes to resource conservation and reduces environmental impact.

## Figures and Tables

**Table 1:** Chemical composition of the materials used (average values).

Component Content, %	SiO_2_	Al_2_O_3_	FeO	MgO	CaO	Cr_2_O_3_	Other Comp.
chamotte	57.1	42.9	-	-	-		residue
slag	63.52	-	33.75	1.55	1.25		residue
clay	43.1	31.0	-	14.2	-	0.3	residue

**Table 2:** Mineral composition of chamotte.

Mineral	Chemical Formula	Content, %
Cristobalite	SiO_2_	29.35
Quartz	SiO_2_	10.90
Mullite	3Al_2_O_3_ ∗ 2SiO_2_	59.8

**Table 3:** Mineral composition of clay.

Mineral	Chemical Formula	Content, %
Kaolinite	Al_2_(Si_2_O_5_)(OH)_4_	70.0
Forsterite	Mg_2_SiO_4_	23
Gibbsite	Al(OH)_3_	3.04
Magnesiochloritoid	MgAl_2_SiO_5_(OH)_2_	2.3
Chromian clinochlore	(Mg_5_Al)(AlSi_3_)O_10_(OH)_8_ (with Cr)	1.7

**Table 4:** Mineral composition of slag.

Mineral	Chemical Formula	Content, %
Quartz	SiO_2_	4.05
Enstatite	MgSiO_3_	3.9
Wollastonite	CaSiO_3_	2.6
Cristobalite	SiO_2_	8.0
Clinoferrosilite	Fe(SiO_3_)	81.55

**Table 5:** Compositions of chamotte mixtures for semi-dry pressing, mass %.

Component	No. 1 (Reference)	No. 2	No. 3	No. 4	No. 5
Coarse filler	53.6	53.6	53.6	53.6	53.6
Chamotte, fraction 3–2 mm	17.6	17.6	17.6	17.6	17.6
Chamotte, fraction 2–0.5 mm	36.0	36.0	36.0	36.0	36.0
Matrix	46.4	46.4	46.4	46.4	46.4
Chamotte, fraction <0.5 mm	36.4	31.4	33.4	28.4	26.4
KBRU-33 clay	10	10	10	15	15
Steelmaking slag	-	5	3	3	5
Total (dry basis)	100	100	100	100	100
Water (above 100%)	5.5–6.5	5.5–6.5	5.5–6.5	5.5–6.5	5.5–6.5

**Table 6:** Results of the conducted tests.

No.	P*_open_*, %	Ρ_every_, g/cm^3^	σ*_aver_*, MPa	Temperature of Deformation Onset, °C
1	22.10	1.99	22	1613
2	24.15	2.30	24	1610
3	22.0	1.90	19	1614
4	21.35	2.01	21	1612
5	21.2	2.02	19	1612

**Table 7:** Comparative slag resistance characteristics of the developed refractory samples.

Sample No.	Appearance of the Tested Samples	Area of Slag–Refractory Interaction, mm^2^	Penetration Depth Δh, mm
1	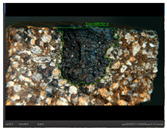	127.24	+0.30
2	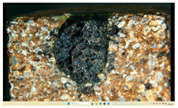	~133	+0.30
3	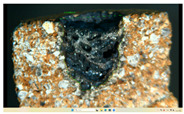	141.97	+0.35
4	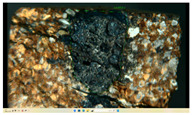	140.64	+0.1 *
5	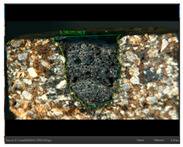	140.77	+0.38

* Note: locally up to 5 mm.

**Table 8:** Mineral composition of the chamotte brick phases (according to XRD data).

Mineral	Chemical Formula	Content, %				
		No. 1	No. 2	No. 3	No. 4	No. 5
Cristobalite	SiO_2_	26.0	26.0	26.0	22.91	23.8
Quartz	SiO_2_	7.69	6.8	6.59	6.49	6.4
Mullite	Al_2_ (Al2.5 Si1.5)	65.9	67.0	67.4	62.2	68.0
Indialite	Mg_2_ (Al3.9 Si5.1 O18)	0.41	0.2	traces	8.4	-
Enstatite, aluminian	(Mg.956 Al.044)	-	traces	-	-	-
Cordierite high	Mg1.91 Fe0.09 Al4	-	-	-	-	1.80

**Table 9:** Quantitative microstructure parameters of the investigated samples.

Sample	1	2	3	4	5
Pore area fraction, %	35.1	26.8	32.6	26.6	29.2
Phase area, ×10^3^ μm^2^	108.8	303.9	356.1	98.1	97.5
Pore area fraction, %	34.0	13.4	20.08	20.52	21.16
Total pore area ×10^3^ μm^2^	43.0	38.9	27.4	21.6	30.4
Average equivalent pore size	14.6	5.7	8.6	8.8	9.1

## Data Availability

The original contributions presented in this study are included in the article. Further inquiries can be directed to the corresponding authors.
